# Rare *De Novo IGF2* Variant on the Paternal Allele in a Patient With Silver–Russell Syndrome

**DOI:** 10.3389/fgene.2019.01161

**Published:** 2019-11-15

**Authors:** Chun-Ling Xia, Yuan Lyu, Chuang Li, Huan Li, Zhi-Tao Zhang, Shao-Wei Yin, Yan Mao, Wen Li, Ling-Yin Kong, Bo Liang, Hong-Kun Jiang, Jesse Li-Ling, Cai-Xia Liu, Jun Wei

**Affiliations:** ^1^Key Laboratory of Maternal-Fetal Medicine of Liaoning Province, Key Laboratory of Obstetrics and Gynecology of Higher Education of Liaoning Province, Liaoning Centre for Prenatal Diagnosis, Research Center of China Medical University Birth Cohort, Department of Gynecology & Obstetrics, Shengjing Hospital affiliated to China Medical University, Shenyang, China; ^2^Basecare Medical Device Co., Ltd., Suzhou, China; ^3^State Key Laboratory of Microbial Metabolism, Joint International Research Laboratory of Metabolic and Developmental Sciences, School of Life Sciences and Biotechnology, Shanghai Jiao Tong University, Shanghai, China; ^4^Department of Pediatrics, The First Affiliated Hospital of China Medical University, Shenyang, China; ^5^Jinxin Research Institute of Reproductive Medicine and Genetics, Jinjiang Maternal and Children’s Health Care Hospital, Chengdu, China

**Keywords:** *IGF2*, *de novo*, splicing variant, Silver–Russell syndrome, whole-exome-sequencing

## Abstract

Silver–Russell syndrome (SRS) is a rare, well-recognized disorder characterized by growth restriction, including intrauterine and postnatal growth. Most SRS cases are caused by hypomethylation of the paternal imprinting center 1 (IC1) in chromosome 11p15.5 and maternal uniparental disomy in chromosome 7 (UPD7). Here, we report on a Chinese family with a 4 year old male proband presenting with low birth weight, growth retardation, short stature, a narrow chin, delayed bone age, and speech delays, as a result of a rare molecular etiology. Whole-exome sequencing was conducted, and a novel *de novo IGF2* splicing variant, NM_000612.4: c.157+5G > A, was identified on the paternal allele. *In vitro* functional analysis by RT-PCR and Sanger sequencing revealed that the variant leads to an aberrant RNA transcript lacking exon 2. Our results further confirm the *IGF2* variant mediates SRS and expand the pathogenic variant and phenotypic spectrum of *IGF2*-mediated SRS. The results indicate that, beyond DNA methylation and UPD7 and *CDKN1C* variant tests, *IGF2* gene screening should also be considered for SRS molecular diagnoses.

## Background

Silver–Russell syndrome (SRS) (MIM number: 180860) is a rare disorder characterized by intrauterine growth retardation accompanied by postnatal growth deficiency. The clinical features include proportionately short stature, normal head circumference, fifth-finger clinodactyly, typical facial features (triangular faces characterized by broad forehead and narrow chin), and limb-length asymmetry potentially resulting from hemihypotrophy with diminished growth of the affected side (Saal, 1993).

SRS is considered an etiologically and clinically heterogeneous disease, thus complicating clinical and molecular diagnosis. An SRS scoring system, the Netchine–Harbison Clinical Scoring System (NH-CSS), was developed for the clinical diagnosis of SRS. This scoring system is recognized in an international consensus statement on the diagnosis and management of SRS (Azzi et al., 2015; Wakeling et al., 2017).

*IGF2*, located in 11p15.5, encodes a member of the insulin family of polypeptide growth factors, which are involved in development and growth (Cassidy and Charalambous, 2018). It is an imprinted gene, expressed only from the paternal allele, and epigenetic changes at this locus are associated with several disorders, including Wilms tumors, Beckwith–Wiedemann syndrome, rhabdomyosarcoma, and SRS (Anderson et al., 1999; Md Zin et al., 2011; Azzi et al., 2014). Previous studies have reported that 30–60% of SRS cases are caused by hypomethylation of the paternal imprinting center 1 (IC1) of chromosome 11p15.5, whereas approximately 10% of cases have maternal uniparental disomy for chromosome 7 (UPD7) (Wakeling et al., 2017). Examination of rare cases has resulted in the identification of pathogenic *CDKN1C* variants as part of the SRS etiology (Wakeling et al., 2017). The molecular etiology of 30–40% of SRS patients with a clinical diagnosis of this condition remains unknown. Recently, a pathogenic *IGF2* variant in a multigenerational family with growth restriction and a phenotype similar to that of SRS was identified (Begemann et al., 2015). Subsequently, five reports revealed SRS-associated *IGF2* variants. However, the variants and phenotypic spectrum remain very limited (Liu et al., 2017; Yamoto et al., 2017; Abi Habib et al., 2018; Poulton et al., 2018).

Here, we report a *de novo IGF2* splicing variant occurring on the paternal allele in an SRS patient born small for his gestational age, and presenting with growth retardation, short stature, and facial abnormalities. The newly identified *IGF2* variant indicates another molecular etiology for SRS beyond the hypomethylation of IC1, UPD7, and *CDKN1C* variants, which should be screened in SRS patients with negative regular molecular diagnosis results.

## Case Presentation

**Figure 1:**
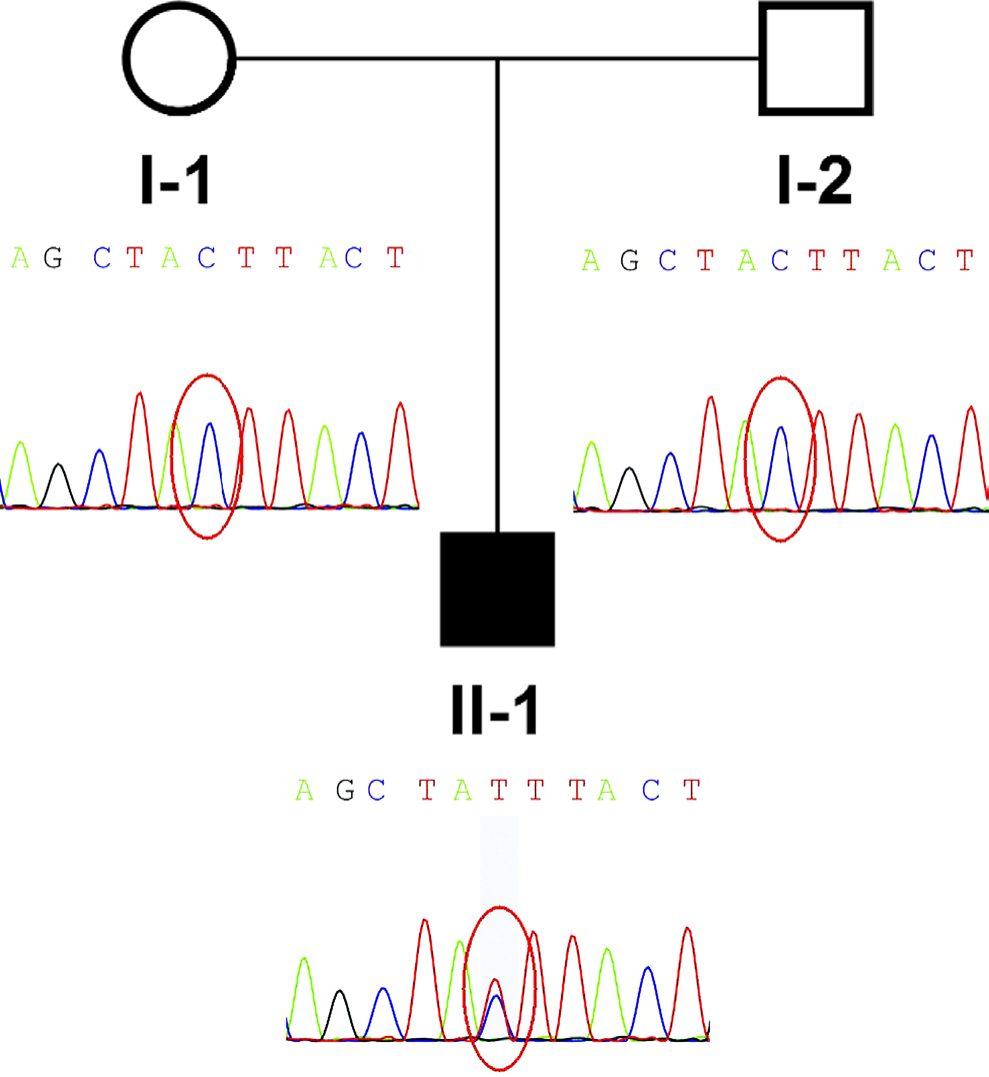
Pedigree and *de novo IGF2* variant identified in the family. The proband (II-1) was the only patient in the family and carried a de novo *IGF2* variant. The parents of the patient were wild type.

The proband is a 4 year old male who is the only child of a Chinese family (**Figure 1**). The parents are non-consanguineous and clinically normal. The heights of the father and mother are 178 cm and 163 cm, respectively. No family members or relatives have developmental delay. During pregnancy, oligohydramnios was observed. The proband was born at 40 weeks’ gestation with a small placenta, and he was relatively small for his gestational age (weight 1,850 g, <3%; length 42 cm, <3%; head circumference 27 cm, <3%). The patient had mild feeding difficulties: he was under-eating and required Enfamil Enfacare. His height and weight at 24 months were 75 cm (<3%) and 7,500 g (<3%), respectively, and his BMI was 10.5 (<3%). In addition, a prominent forehead, fifth-finger clinodactyly, a triangular face, micrognathia, low-set ears, delayed bone age, low muscle mass, delayed motor, and speech development were identified (**Table 1**). No other obvious abnormalities were found. According to growth restriction, the patient was subjected to GH therapy, which was efficacious; the patient grew 3 cm after 16 GH injections administered over the course of 114 days.

**Table 1:** *IGF2* genetic defects and clinical features of patients with Silver-Russell syndrome.

Phenotype	Present Case	Begemann et al., 2015				Yamoto et al., 2017	Deguo Liu et al., 2017	Walid Abi Habib et al., 2018		Cathryn Poulton et al., 2018
		Patient 1	Patient 2	Patient 3	Patient 4			Case 1	Case 2	
Country of Origin	China	Germany				Japan	China			Australian Aboriginal
Genetic Defect	NM_000612.4: c.157+5G > A	NM_001127\ 598.2:c. 191G > A	NM_00112 7598.2: c.191G > A	NM_00112 7598.2: c.191G > A	NM_00112 7598.2: c.191G > A	NM_000612.5:c.110_117delinsAGGTAA	NM_000612: c.101G > A	NM_0006 12.5:c.78C > G	NM_000612.5: c.158_159dup	NM_000612.5: c.157+3A > C
Source of Variation	Paternal allele, *De novo*	Paternal	Paternal	Paternal	Paternal	Paternal allele, *De novo*	Paternal allele, *De novo*	*De novo*	*De novo*	Paternal allele, *De novo*
Gender	M	M	F	M	F	M	M	M	F	F
Height of Father	178	178	178	182	150.5 (patient1)	173				
Height of Mother	163	163	163	163	147	167				
Gestational-age	40	43	36	36	35	31	37	32	29	32
Small for Gestational Age	+	+	+	+	+	+	+	+	+	+
Head Circumference at Birth	27					NA	33	27/–2.13	23.5/–2.3	28
Prominent Forehead(1–3-years-old)	+	+	+	−	+	+	+	+	+	+
Body Asymmetry	−	−	−	−	−	−	+	−	−	
Feeding Difficulties and/or Low BMI	+	+	+	−	+	+	+	+	+	+
Postnatal Growth Failure	+	+	+	+	+	+	+	+	+	+
Relative Macrocephaly	+	+	+	+	+	+	+	+	+	+
Hypoplastic Placenta	+					+				
Oligohydramnios	+		+			NA				
Triangular Face	+	+	+	+	+	+	+	+	+	
Micrognathia	+	+	+	−	+	+	+			+
Cleft Palate	−					+				
Low-set Ears	+	+	+	+	+	+	+			
Hearing Impairment	−					Borderline hearing impairment				
Low Muscle Mass	+									
Clinodactyly	+		+			+				+
Ectrodactyly	−					+				
Polydactyly	−					+				
Syndactyly	−					+				
Shoulder Dimples	−									+
Bone Age Delay	+	+	+	+	NA					
Low-intelligence	−	+	+	−	−					
Hypospadia	−			+	−	+				
Abnormal Scrotum	−					+				
Cryptorchidism	−			+		+				
Microphallus	−					+				
Heart defect	−	+	+		+	+	+			+
Brain	−									Posterior periventricular white matter loss and periventricular leukomalacia
Motor Delay	+	+	+	−	+		−			+
Speech Delay	+	+	+	−	−	−				
Other Abnormity	NA									
GH Response	+	+	+	+	+	NA	+	+	+	+

According to the NH-CSS, the patient was diagnosed with SRS. Because the patient was born small for his gestational age and did not show body asymmetry, we initially speculated that there might be a genetic, rather than an epigenetic, defect causing the patient’s phenotype. Peripheral blood samples from the family members and a healthy control member were collected. The family members and the healthy control member provided written informed consent to participate in the study. This study was approved by the ethics committee of Shengjing Hospital of China Medical University. The patient’s samples were subjected to next generation sequencing-based copy number variation assays, and WES trio testing was performed according to our rare disorder diagnosis process.

## Whole-Exome Sequencing

Low depth whole genome sequencing based on next generation sequencing (NGS) and analysis of copy number variant was performed in the family. TMAP software (Version 4.6), Picard software (Version 2.18.17), and the analytic method of LOWESS regression and circular binary segmentation were used to analyze copy number variant. Only a single maternal 0.41Mb duplication (17q11.2: 28920000-29330000) was found. Only RNF135 within this interval has been previously associated with abnormal phenotype, including overgrowth, mild learning disability, strabismus, dysmorphism, and advanced bone age (Douglas et al., 2007). This variant alone could not explain the phenotype of the patient. Subsequently, whole-exome sequencing (WES) of the patient-parent trio was conducted (**Figure 1 II-1, I-1, I-2**). WES of the family members yielded more than 14.1 Gb of data at 92× the average depth with an overall coverage greater than 97.65%. A total of 675 variants with minor allele frequencies of <0.01 in any of the study-associated databases (dbSNP, Exome Aggregation Consortium, 1000 Genomes Project, HapMap project, and in-house database) were selected for further analysis. Variant analysis was performed based on an autosomal recessive inheritance model, including compound heterozygous and homozygous variants, or based on an autosomal dominate inheritance model, including heterozygous variants. In addition, *CDKN1C* and *IGF2* gene variants were considered in the analysis. No *CDKN1C* variant was found, whereas a *de novo IGF2* gene variant, NM_000612.4: c.157+5G > A, was identified. Because *IGF2* is a paternally expressed gene, *IGF2* gene defects on the paternal chromosome could induce disease. A heterozygous SNP rs3213225 (NM_000612.4:c.157+61C > T) adjacent to c.157+5G > A was additionally located in the same read. This SNP was paternal in origin, and the mother did not harbor this variant. Finally, the presence of the variant was verified by Sanger sequencing.

## *In Vitro* Functional Analysis

**Figure 2:**
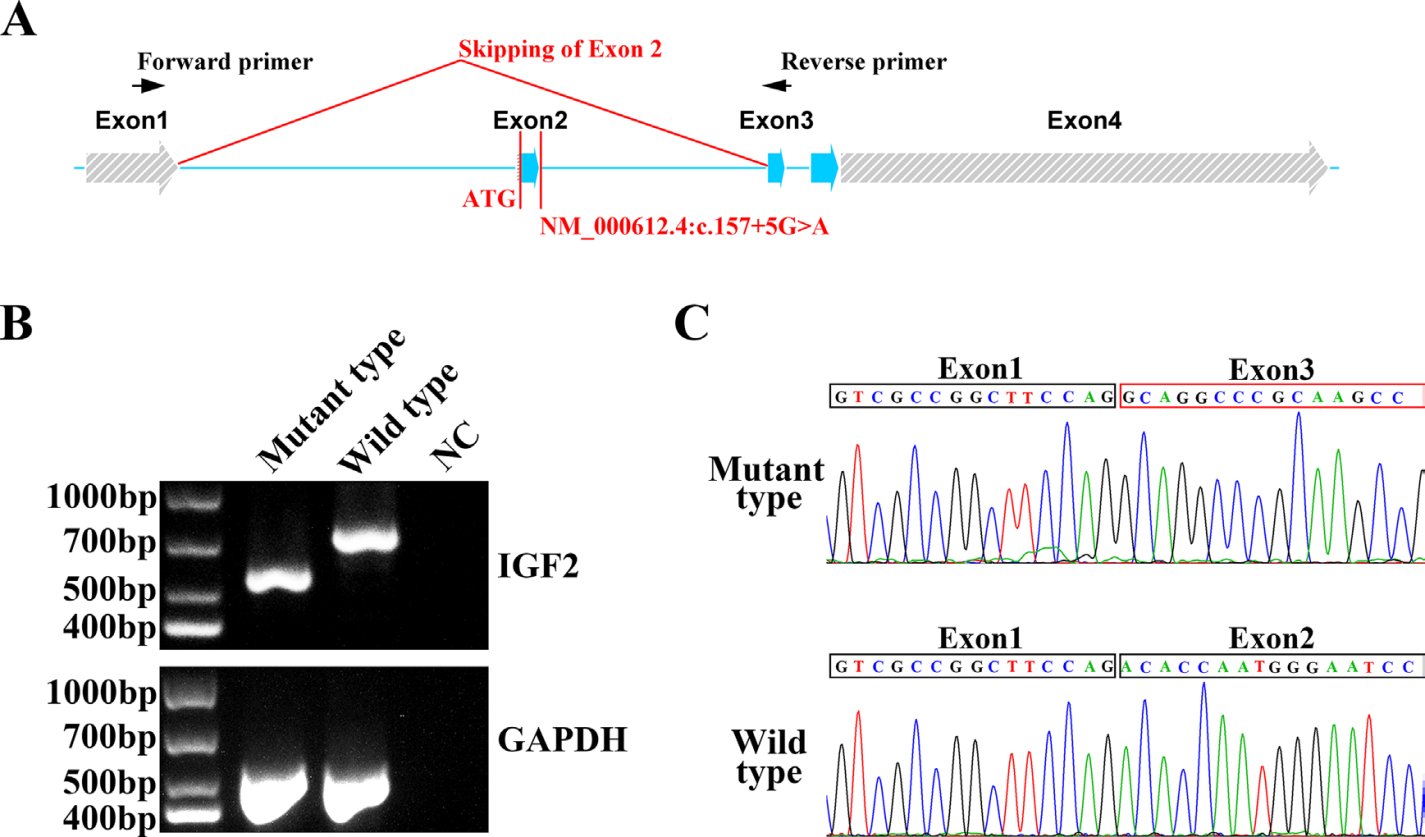
*In vitro* functional analysis of *IGF2* splicing variant. **(A)** The *IGF2* gene was consisted with four exons. The NM_000612.4: c.157+5G>A variant was in intron 2. The AUG initiation codon of *IGF2* was in exon 2. The RT-PCR primers were designed for exon 1 and exon 3. **(B)** Agarose gel electrophoresis of the RT-PCR products from the proband and the healthy control. The PCR of the mutant type mRNA from the proband produced a truncated electrophoretic band of about 600 bp. GAPDH was used as an internal control. **(C)** Sanger sequencing of mutant type and wild type PCR products. Exon 2 skipping was clearly observed in mutant type mRNA, while the normal mRNA sequence was observed in the wild type.

To confirm the function of the newly identified variant, we conducted *in vitro* functional analysis with RT-PCR and Sanger sequencing. Total mRNA of the proband and a healthy control was extracted from blood, and cDNA was synthesized from the total mRNA by performing reverse transcription with a modified oligo(dT) primer. PCR was conducted with a pair of primers binding exons 1 and 3 (**Figure 2A**). The wild type mRNA produced the expected PCR product of 700 bp. However, the mRNA of the proband yielded a shorter electrophoretic band of 600 bp, indicating a truncated mRNA (**Figure 2B**). Sanger sequencing of the RT-PCR products from the healthy control and the patient indicated that the NM_000612.4: c.157+5G > A variant led to the skipping of exon 2 in the *IGF2* gene (**Figure 2C**). This exon skipping resulted in a loss of the coding sequence for exon 2 and, more importantly, the loss of the AUG initiation codon.

## Discussion

The estimated incidence of SRS is 1:30,000 to 1:100,000. Nevertheless, the incidence may be underestimated because of limitations in molecular diagnosis techniques (Wakeling et al., 2017). The major molecular etiology of SRS is based on loss of methylation on chromosome 11p15 and maternal uniparental disomy of chromosome 7, which account for 30–60% and 5–10% of molecular diagnoses in SRS patients, respectively (Wakeling et al., 2017). The molecular etiology of the remaining undiagnosed patients warrants further investigation. A maternal *CDKN1C* gain-of-function variant has been reported as a rare cause of SRS (Eggermann et al., 2014). Recently, five reports have identified *IGF2* loss-of-function variants as new contributors to the molecular etiology of SRS, on the basis of studies including individuals of German, Japanese, Chinese, and Australian Aboriginal descent (**Table 1**) (Begemann et al., 2015; Liu et al., 2017; Yamoto et al., 2017; Abi Habib et al., 2018; Poulton et al., 2018). In our SRS patient from China, we found a novel *IGF2* splicing variant, NM_000612.4: c.157+5G > A, which is located on the paternal allele. According to *in vitro* functional analysis by RT-PCR, the variant results in skipping of exon 2, which contains the *IGF2* initiation codon. The variant is absent from control databases including gnomAD (http://gnomad.broadinstitute.org/), ExAC (http://exac.broadinstitute.org/), and the 1000 Genomes project (http://www.internationalgenome.org/). According to the ACMG guidelines, the NM_000612.4: c.157+5G > A variant is described as PVS1 (initiation codon or single or multi-exon deletion), PS2 (*de novo*), and PM2 (absent from controls), and therefore is classified as pathogenic (Richards et al., 2015). The SNP rs3213225 (NM_000612.4:c.157+61C > T) was located in the deep intron region, with a relatively high minor allele frequency (0.449) in 1,000 genomes. The SNP is in cis with the the c.157+5G > A *de novo* mutation, and 56 nt away from it. We consider that rs3213225 has no obvious effects on splicing, and it is very unlikely to be related to phenotype in this case. While it is very unlikely that the common cis polymorphism rs3213225 has any impact on the splicing defect caused by the *de novo* mutation, we cannot positively exclude that possibility. The six previously identified variants include missense, frameshift, stop-gain, and splicing variants (**Table 1**). No major variation pattern appears to exist for *IGF2*.

SRS, first described by Silver et al. and Russell (Silver et al., 1953; Russell, 1954), is mainly characterized by small size for gestational age, postnatal short stature, body asymmetry, relative macrocephaly, a prominent forehead, and feeding difficulties. A patient with more than four of the major characteristic features would be clinically diagnosed with SRS (Azzi et al., 2015). Other phenotypic characteristics often observed in SRS patients include a downturned mouth, clinodactyly of the fifth finger, shoulder dimples, syndactyly of the 2/3 toes, low muscle mass, a prominent heel, autism, and diagnosed cognitive disabilities (Wakeling et al., 2017). However, the phenotypes of *IGF2* gene variants associated with SRS may be different. On the basis on the cases linked to *IGF2* variants (**Table 1**), we conclude that most of these patients do not have body asymmetry (nine in ten patients), a major classification condition according to the NH-CSS for SRS clinical diagnosis. This difference may be a primary condition for diagnosis of SRS caused by epigenetic changes or the presence of an *IGF2* variant. This finding, as well as the case summary, may be useful for the molecular diagnosis of SRS.

In addition, low muscle mass (one in ten patients) was observed in our patient. This finding has not previously been reported in patients with SRS caused by *IGF2* variant (**Table 1**). Hypoplastic placenta (two in ten patients), oligohydramnios (two in ten patients), bone age delay (four in ten patients), low intelligence (two in ten patients), and speech delay (three in ten patients) were uncommon phenotypes in patients with SRS (**Table 1**). Apart from small size for gestational age, postnatal short stature, body symmetry, relative macrocephaly, it can be concluded that low muscle mass, as described in this report, in addition to the previously described phenotypes, are the second characteristics of SRS caused by *IGF2* variants.

A different variant reported in an Australian Aboriginal family, NM_000612.5:c.157+3A > C, also causes skipping of exon 2 (Poulton et al., 2018). Despite the same *in vitro* functional results, the phenotypes of the two patients were not fully consistent. Hypoplastic placenta, oligohydramnios, low muscle mass, bone age delay, and speech delay were observed in only our patient. Shoulder dimples, heart defects, posterior periventricular white matter loss, and periventricular leukomalacia were found only in the Australian Aboriginal patient. These results indicate the clinical heterogeneity of the *IGF2* variant causing SRS. In addition, the phenotypes of SRS caused by *IGF2* variants may differ according to ethnicity. This viewpoint has previously been described by Poulton et al.(Poulton et al., 2018). To date, all nine patients treated with growth hormone (GH) presented positive effects (**Table 1**), thus indicating that this therapy is an effective treatment for *IGF2*-associated SRS.

To date, four patients from a multigenerational family and five patients with *de novo IGF2* gene variants have been reported in only five reports worldwide (Begemann et al., 2015; Liu et al., 2017; Yamoto et al., 2017; Abi Habib et al., 2018; Poulton et al., 2018). Herein, we present the second reported case of SRS caused by an *IGF2* splicing variant, in a Chinese individual, and identified a novel pathogenic *IGF2* splicing variant. We suggest that an *IGF2* gene variant should be considered in the molecular diagnosis process, especially when body asymmetry is not observed in the clinical diagnosis of an SRS patient. In addition, we identified low muscle mass in the patient, a previously unreported phenotype, thus expanding the phenotypic spectrum of *IGF2*-associated SRS. In a comparison in two different ethnic groups yielding the same molecular result of skipping of exon 2, we observed several different phenotypes. These findings indicate the heterogeneity and ethnic differences of SRS caused by *IGF2* variants. However, owing to the limited number of reported cases, further investigation is necessary to verify the phenotypic spectrum, typical characteristics, and clinical therapy in *IGF2*-variant-associated SRS. Collectively, we hope that our findings will facilitate the clinical and molecular diagnosis of SRS.

## Ethics Statement

This study was approved by the Medicine Ethics Committee of Shenjing Hospital of China Medical University. Written informed consent to participate in this study was provided by the participants’ legal guardian/next of kin.

## Author Contributions

C-LX and JW conceived and designed the experiments. YL, CL, and C-XL helped in patient workup and recruitment of the patients and their family members. HL, Z-TZ, and S-WY performed the experiment. YM, WL, L-YK, and BL helped in genetic analysis. H-KJ, JL-L, and JW wrote the paper.

## Funding

This work was supported by the National Key Research and Development Program of China (No. 2016YFC1000408 & No. 2018YFC1002900) and the Natural Science Foundation of China (No. 81701462).

## Conflict of Interest

Author YM, WL, and LY-K were employed by company Basecare Medical Device Co., Ltd., Suzhou.

The remaining authors declare that the research was conducted in the absence of any commercial or financial relationships that could be construed as a potential conflict of interest.

## References

[B1] Abi Habib W. Brioude F. Edouard T. Bennett J. T. Lienhardt-Roussie A. Tixier F. (2018). Genetic disruption of the oncogenic HMGA2-PLAG1-IGF2 pathway causes fetal growth restriction. Genet. Med. 20 (2), 250–258. 10.1038/gim.2017.105 28796236 PMC5846811

[B2] Anderson J. Gordon A. Pritchard-Jones K. Shipley J. (1999). Genes, chromosomes, and rhabdomyosarcoma. Genes Chromosomes Cancer 26 (4), 275–285. 10.1002/(sici)1098-2264(199912)26:4<275::aid-gcc1>3.0.co;2-3 10534762

[B3] Azzi S. Abi Habib W. Netchine I. (2014). Beckwith-Wiedemann and Russell-Silver Syndromes: from new molecular insights to the comprehension of imprinting regulation. Curr. Opin. Endocrinol. Diabetes Obes 21 (1), 30–38. 10.1097/MED.0000000000000037 24322424

[B4] Azzi S. Salem J. Thibaud N. Chantot-Bastaraud S. Lieber E. Netchine I. (2015). A prospective study validating a clinical scoring system and demonstrating phenotypical-genotypical correlations in Silver-Russell syndrome. J. Med. Genet. 52 (7), 446–453. 10.1136/jmedgenet-2014-102979 25951829 PMC4501172

[B5] Begemann M. Zirn B. Santen G. Wirthgen E. Soellner L. Buttel H. M. (2015). Paternally inherited IGF2 mutation and growth restriction. N. Engl. J. Med. 373 (4), 349–356. 10.1056/NEJMoa1415227 26154720

[B6] Cassidy F. C. Charalambous M. (2018). Genomic imprinting, growth and maternal-fetal interactions. J. Exp. Biol. 221 (Pt Suppl 1), jeb164517. 10.1242/jeb.164517 29514882

[B7] Douglas J. Cilliers D. Coleman K. Tatton-Brown K. Barker K. Bernhard B. (2007). Mutations in RNF135, a gene within the NF1 microdeletion region, cause phenotypic abnormalities including overgrowth. Nat. Genet. 39 (8), 963–965. 10.1038/ng2083 17632510

[B8] Eggermann T. Binder G. Brioude F. Maher E. R. Lapunzina P. Cubellis M. V. (2014). CDKN1C mutations: two sides of the same coin. Trends Mol. Med. 20 (11), 614–622. 10.1016/j.molmed.2014.09.001 25262539

[B9] Liu D. Wang Y. Yang X. A. Liu D. (2017). *De Novo* mutation of paternal igf2 gene causing silver-russell syndrome in a sporadic patient. Front. Genet. 8, 105. 10.3389/fgene.2017.00105 28848601 PMC5550680

[B10] Md Zin R. Murch A. Charles A. (2011). Pathology, genetics and cytogenetics of Wilms’ tumour. Pathol. 43 (4), 302–312. 10.1097/PAT.0b013e3283463575 21516053

[B11] Poulton C. Azmanov D. Atkinson V. Beilby J. Ewans L. Gration D. (2018). Silver Russel syndrome in an aboriginal patient from Australia. Am. J. Med. Genet. A. 176 (12), 2561–2563. 10.1002/ajmg.a.40502 30152198

[B12] Richards S. Aziz N. Bale S. Bick D. Das S. Gastier-Foster J. (2015). Standards and guidelines for the interpretation of sequence variants: a joint consensus recommendation of the American College of Medical Genetics and Genomics and the Association for Molecular Pathology. Genet. Med. 17 (5), 405–424. 10.1038/gim.2015.30 25741868 PMC4544753

[B13] Russell A. (1954). A syndrome of intra-uterine dwarfism recognizable at birth with cranio-facial dysostosis, disproportionately short arms, and other anomalies (5 examples). Proc. R. Soc Med. 47 (12), 1040–1044. 13237189

[B14] Saal H. M. (1993). Russell-Silver Syndrome, in GeneReviews((R)). Eds. Adam M. P. Ardinger H. H. Pagon R. A. Wallace S. E. Bean L. J. H. Stephens K. Amemiya A. (Seattle (WA)): University of Washington, Seattle. 20301499

[B15] Silver H. K. Kiyasu W. George J. Deamer W. C. (1953). Syndrome of congenital hemihypertrophy, shortness of stature, and elevated urinary gonadotropins. Pediatr. 12 (4), 368–376. 10.1016/S0022-3476(53)80440-3 13099907

[B16] Wakeling E. L. Brioude F. Lokulo-Sodipe O. O’Connell S. M. Salem J. Bliek J. (2017). Diagnosis and management of Silver-Russell syndrome: first international consensus statement. Nat. Rev. Endocrinol. 13 (2), 105–124. 10.1038/nrendo.2016.138 27585961

[B17] Yamoto K. Saitsu H. Nakagawa N. Nakajima H. Hasegawa T. Fujisawa Y. (2017). *De novo* IGF2 mutation on the paternal allele in a patient with Silver-Russell syndrome and ectrodactyly. Hum. Mutat. 38 (8), 953–958. 10.1002/humu.23253 28489339

